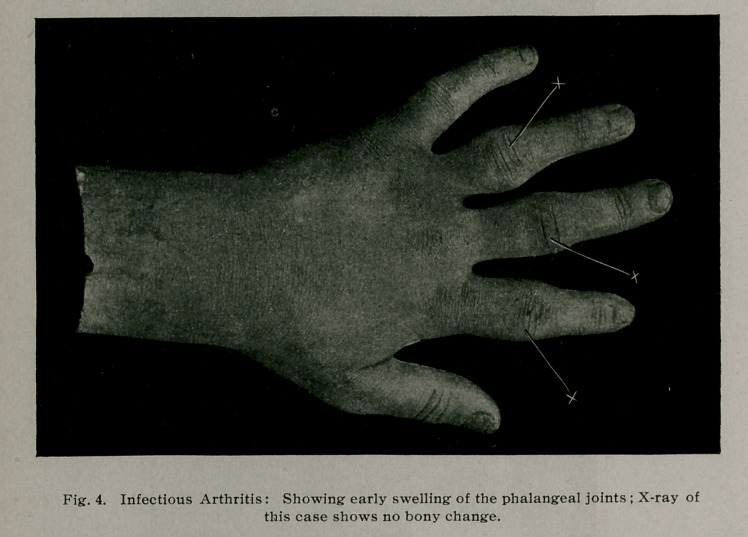# Non-Tubercular Joint Lesions

**Published:** 1906-01

**Authors:** Roland O. Meisenbach

**Affiliations:** Buffalo, N. Y.


### BUFFALO MEDICAL JOURNAL.

VOL. LXI. JANUARY, 1906. No. 6

### ORIGINAL COMMUNICATIONS.

### Non-Tubercular Joint Lesions.

By ROLAND O. MEISENBACH, M. D., Buffalo, N. Y.

FROM the study of a large number of non-tubercular joint cases, over a long period, it has clearly been shown by Dr. Goldthwait and his associates, that the joint diseases commonly called by the names of chronic rheumatism, gout, arthritis deformans, gouty rheumatism and many others, are not one and the same disease, but are distinct diseases, both clinically and pathologically. The different types run an altogether different course and yield to essentially different treatment.

The first type, *Chronic Villous Arthritis,* in its uncomplicated form, is what is generally spoken of as the dry, hyperemic or relaxed joint and is really a villous arthritis; it is characterised in its simplest form as a local process, but may or may not complicate the other types of joint disease. It is seen most marked in the knee, but may be present in any of the other joints. It is characterised clinically, at first, by a swelling of the affected joints, with local pain and tenderness. In both active, and passive motion, there is crepitus or creaking over the joint, and quite often sudden check of motion, or locking, takes place.The joint structures are usually relaxed and a flabby condition of the mucous membrane exists; this flabby membrane protrudes into the joint, and may obstruct, mechanically, its action. The rubbing together of the folds of flabby synovial membrane, causes crepitation on motion, and the continued use, or irritation of this, causes it to be passively congested, thickened, and as a result, villi or fringes form. If, however, the joint continues to be used, this passively hyperemic membrane undergoes a degeneration in character, and the villi formed give rise to tabs, which may project into the joint and become detached, consequently acting as foreign bodies,—"Joint Mice." The fluid in the joint may be normal or in excess. The process is not migratory but may exist simultaneously in more than one joint.

Villous Arthritis may be due to joint strain or to general diathesis; often flat-foot and genu valgum or any other joint strain are the causes, but more frequently a general lowered vitality of the individual.

The treatment is early stimulation, locally, with partial fixation of the joint by a bandage, and if symptoms of synovial changes are present, or if the condition is due to faulty attitude, operation or the correction of the faulty attitude, offers immediate relief.

The second type, *Atrophic or Rheumatoid Arthritis*, is, as its name implies, chiefly characterised, pathologically, by early atrophy of the articular and periarticular structures. It is a pro-

Fig, I. Atrophic Arthritis: Showing apparent swelling, but real atrophy and telescoping of the metacarpo-phalangeal joints.

gressive disease, first attacking one joint, or a group of joints, and then involving others. Although its etiology has not yet been definately determined, it seems, from my observations in large clinics, that it coexists with nervous exhaustion and has a predilection for the female sex from sixteen years upward. The disease is ushered in by an early swelling of the affected joints with a subsequent early atrophy of the articular cartilage cells, which, as the swelling subsides, becomes marked. At first the articular cartilage cells atrophy, later the bones, especially the articular ends and shafts, and finally the soft structures around the joints (Fig. 2); even the skin over the joint, which appears glossy, has undergone atrophy (Fig. 1). Villous Arthritis often accompanies the early stage of atropic arthritis and as a symptom we may get crepitation and excess of fluid, but this soon disappears as the atrophy increases. The disease is seen, usually, to appear in the metacarpo-phalangeal joints, and in course of time, involves the knees, shoulders, and other joints if left untreated. The ankylosis in the case of rheumatoid arthritis is a fibrous one caused by the atrophy of the articular and periarticular structures. This disease if left to run its course, leads to marked crippling (Fig. 1). The blood shows little change during the entire course of the disease.

The treatment is constitutional by giving tonics and by forced feeding, by the proper ingestion of fats, such as Codliver oil, butter, or pure olive oil. Meats should be given very abundantly, whether light or dark makes little difference. The anabolic and katabolic conditions of the patient should be brought to the normal and the patient should have mental and physical rest as well. The affected joints should at first, receive rest and later active and passive motion. As the disease advances the patient should be instructed to use the joints and together with stimulation by massage and hot formentations. the circulation, consequently the atrophic condition is improved. If the disease has gone so far that ankylosis or deformity exists, they can be benifited by operative methods.

The third type, the *Hypertropic or Osteo-Arthritis,* may be a local or general process; it is characterised by the thickening of the edges of the articular cartilages, forming ridges or nodes which subsequently undergo ossification and interfere with joint motion. As the process extends the cartilage at the points of pressure is absorbed, replaced by bone and a true bony hypertrophy exists (Fig. 3.) This type of disease is sometimes well illustrated by the specimens of "Ossified Men" which we occasionally see. The symptoms of hypertrophic arthritis are chiefly due to the pressure of the hypertrophied bone on the nerves, either by pushing them out of their normal place or by narrowing their channel. The rigidity or the ankylosis of the affected joints is chiefly due to the increased amount of ostoid tissue within a given space and thereby preventing motion, but in severe cases actual fusion of the bone may take place. It is in this disease that we get so much referred pain, and to any part of the body; when the lumbar and sacral nerves are pressed upon, it is often taken for sciatica.

The real cause of the disease is not yet known, but undoubtedly it can often be traced back to exposure to cold, wet, or overexertion, and frequently to injury.

The disease is not a progressive one, to the extent that the atrophic type is, and runs a more irregular course and with proper treatment, subsides. It is usually first noticed in the spine

Fig. 2. Atrophic Arthritis: Showing atrophy of the cartitage and the beginning atrophy of the bones with telescoping of the articular ends of the bone. (Goldthwait.)

or distal phalangeal joints (Fig. 3). The blood so far as has been determined shows no marked change.

The treatment consists of early fixation of the joints affected, so as to prevent irritation of the parts. The eliminative functions should be regulated and the general health improved. When hypertrophied bone interferes with joint motion, it should be removed.

The fourth class, one of the most common and far-reaching, is the *Infectious Arthritis;* this may be an acute or chronic arthritis but it is usually this type which is referred to as chronic rheumatism. It is an infectious inflammatory process, involv-

Fig. 3. Hypertrophic Arthritis: Showing ossification of intervertebral discs, and the over-lapping of hypertrophied bone. (C. F. Palmer.)

ing the joints and surrounding soft parts, and is caused by the entrance into the joint, either by the micro-organism its self, or by the toxins therof: the pneumococcus, streptococcus. typhoid bacillus, bacillus of influenza etc., may all cause this type in a mild or severe form. The disease may manifest itself as mon-articular or poly-articular (Fig. 4) but in this it differs from the atropic and hypertropic in so much that, in this type, the affected joints usually show their afinity early and in a short time. The severity of the disease depends on the initial organism and the extent of inflammation present. The capsule may become thickened or pus may form with resulting destruction of the joint, as is sometimes the case with the gonococcus.

The disease is a toxemia and associated with the joint symptoms, other general symptoms, such as, glandular enlargement throughout the body, septic temperature and cardiac lesions may exist. The blood is at first normal but as the disease continues, a secondary anemia presents itself.

Fig. 4. Infectious Arthritis: Showing early swelling of the phalangeal joints; X-ray of this case shows no bony change.

The treatment is that of toxemia, combined with local procedures; the septicemia should be treated by tonics, good food and the eliminative functions should receive attention. Much water should be ingested. The local treatment consists of resting the affected joints, together with stimulation, as the disease takes a more chronic form. In cases where the joint shows evidence of pus formation, it should be opened and irrigated.

The fifth type, *Chronic Gout,* is a rare disease and differs essentially from the atrophic or hypertrophic arthropatheis in so much that in chronic gout, the bone is generally attacked secondarily and that crystals of sodium urate are deposited in the periarticular structures; also in gout, the shafts of the bones are slowly absorbed; the absorption is more local, at first, showing definite punched out areas and later involving the entire bone. The large toe and often the thumb are first attacked. They become swollen, very painful, and eventually deformed. This disease admits to much further study and at present the treatment is symptomatic.

### RECAPITULATION

1. Villous Arthritis is a local process, affecting chiefly the synovial membrane of the joint.

2. Rheumatoid or Atrophic Arthritis is a progressive disease, running a definite course, with early atrophy of the joint structures, resulting in marked crippling if left untreated.

3. Hypertrophic or Osteo-Arthritis, is irregular in its course, characterised by true hypertrophy of the bone and ossification of the articular cartilage and the ligaments.

4. Infectious Arthritis may be caused by any of the microorganisms of their toxins, may manifest itself as mono- or polyarticular and results in thickening of the tissues with resulting ankylosis.

5. Chronic Gout, is a comparatively rare disease, showing deposits of crystaline substances in the periarticular structures, and resulting in the absorption of the bone, which begins as localised punched out areas, first affecting the diaphysis and finally the entire bone.

140 Allen Street.

## Figures and Tables

**Fig. I. f1:**
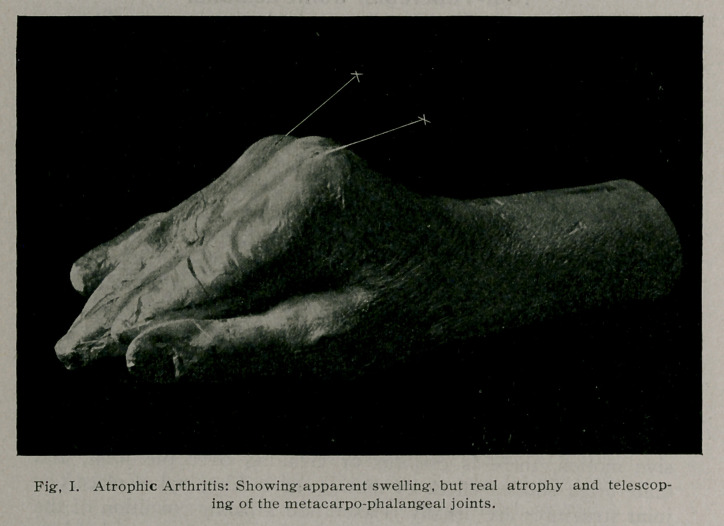


**Fig. 2. f2:**
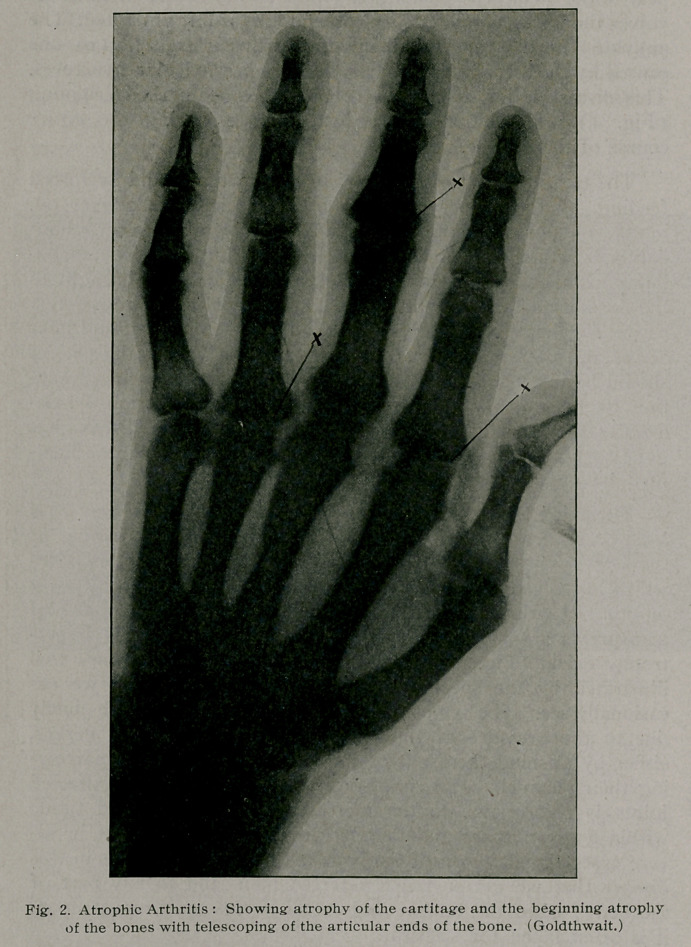


**Fig. 3. f3:**
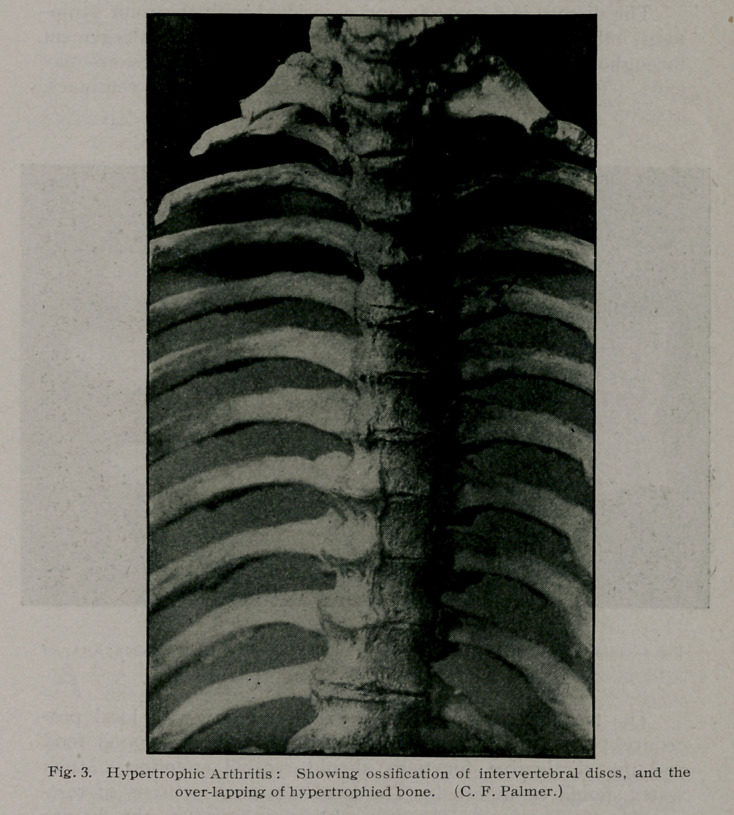


**Fig. 4. f4:**